# Protocol for the Inroads Study: A Randomized Controlled Trial of an Internet-Delivered, Cognitive Behavioral Therapy–Based Early Intervention to Reduce Anxiety and Hazardous Alcohol Use Among Young People

**DOI:** 10.2196/12370

**Published:** 2019-04-12

**Authors:** Lexine A Stapinski, Katrina Prior, Nicola C Newton, Mark Deady, Erin Kelly, Briana Lees, Maree Teesson, Andrew J Baillie

**Affiliations:** 1 Centre of Research Excellence in Mental Health and Substance Use National Drug & Alcohol Research Centre University of New South Wales Sydney Australia; 2 The Matilda Centre University of Sydney Sydney Australia; 3 Black Dog Institute University of New South Wales Sydney Australia; 4 Faculty of Health Sciences University of Sydney Sydney Australia

**Keywords:** alcohol abuse, alcohol-related disorders, anxiety, comorbidity, early medical intervention, cognitive behavioral therapy, young adult

## Abstract

**Background:**

The transition to adulthood is a unique developmental period characterized by numerous personal and social role changes and increased opportunities for alcohol consumption. Using alcohol to cope with anxiety symptoms is commonly reported, and young people with anxiety are at a greater risk of hazardous alcohol use and progression to alcohol use disorder. Anxiety and alcohol use tend to fuel each other in an exacerbating feed-forward cycle, leading to difficult-to-treat chronic problems. The peak in onset of anxiety and alcohol disorders suggests this developmental window represents a promising opportunity for early intervention before these problems become entrenched.

**Objective:**

This study aims to evaluate the efficacy of the *Inroads* program, a therapist-supported, internet-delivered early intervention for young adults that targets alcohol use, anxiety symptoms, and the interconnections between these problems.

**Methods:**

A randomized controlled trial will be conducted nationally among young Australians (aged 17-24 years) who experience anxiety symptoms and drink alcohol at hazardous or harmful levels. Participants will be individually randomized on a 1:1 basis to receive the *Inroads* intervention or assessment plus alcohol guidelines. Participants randomized to the *Inroads* intervention will receive access to 5 Web-based cognitive behavioral therapy (CBT) modules and weekly therapist support via email and/or phone. The primary outcome assessment will be 8 weeks post baseline, with follow-up assessment 6 months post baseline to determine the sustainability of the intervention effects. Primary outcomes will be the total number of standard drinks consumed in the past month (assessed by the Timeline Follow-Back procedure), severity of alcohol-related harms (assessed by the Brief Young Adult Alcohol Consequences Questionnaire), and anxiety symptoms across multiple disorders (assessed by the Generalized Anxiety Disorder-7). Secondary outcomes will include alcohol outcome expectancies; functional impairment and quality of life; and symptoms of social anxiety, anxious arousal, and depression. Results will be analyzed by intention-to-treat using multilevel mixed effects analysis for repeated measures.

**Results:**

The study is funded from 2017 to 2020 by Australian Rotary Health. Recruitment is expected to be complete by late-2018, with the 6-month follow-ups to be completed by mid-2019. Results are expected to be published in 2020.

**Conclusions:**

The study will be the first to evaluate the benefits of a youth-focused early intervention that simultaneously targets anxiety and hazardous alcohol use. By explicitly addressing the interconnections between anxiety and alcohol use and enhancing CBT coping skills, the *Inroads* program has the potential to interrupt the trajectory toward co-occurring anxiety and alcohol use disorders. The Web-based format of the program combined with minimal therapist support means that if effective, the program could be widely disseminated to reach young people who are not currently able or willing to access face-to-face treatment.

**Trial Registration:**

Australian New Zealand Clinical Trials Registry ACTRN12617001609347; https://www.anzctr.org.au/Trial/Registration/TrialReview.aspx?id=372748&isReview=true (Archived by WebCite at http://www.webcitation.org/77Au19jmf)

**International Registered Report Identifier (IRRID):**

DERR1-10.2196/12370

## Introduction

### Background

The transition from adolescence to early adulthood is a unique and important developmental period characterized by numerous personal and social role changes, including new relationships and living arrangements, increased independence, and pursuit of employment and/or higher education [1]. Young adulthood also marks a period of increased vulnerability for the onset of both anxiety and alcohol use disorders [2-5]. Up to 1 in 5 young people aged between 16 and 29 years report an anxiety disorder in the preceding 12 months (12.2%-22.3%) [6-9]. Experiencing anxiety symptoms at this important developmental stage affects the way a young person adjusts to changing life circumstances and can make it more difficult to form new friendships and navigate the challenges, increasing life demands and stressors that emerge [10]. Young adulthood is also characterized by increased availability and opportunities for alcohol use, coinciding with a change to the legal of drinking at age 18 years in many countries including Australia. Within a 12-month period, nearly 1 in 2 (42.0%) young adults aged 18 to 24 years report consuming alcohol at high-risk levels (ie, 5 or more standard drinks on a single occasion at least once a month) and approximately 1 in 6 (15.3%) at very high–risk levels (ie, 11 or more standard drinks on a single occasion at least once per year) [11]. Intoxication, particularly at this age, can have significant health, legal, social, and financial consequences, including progression to alcohol use disorder [1]. Patterns of alcohol use established at this age are linked to outcomes later in adulthood, including risk of chronic alcohol use problems and a range of physical, social, and mental health consequences [12]. Despite the profound potential impact, less than 1 in 4 young people with a mental health or substance use disorder will seek help for these problems [2,13]. For young people, common barriers to seeking treatment include fear of judgment or stigma and practical constraints including difficulty accessing treatment at a convenient time or location [14].

### Interrelationship Between Anxiety and Alcohol Use

Anxiety has been consistently associated with increased risk of hazardous alcohol use and alcohol use disorder [15,16]. In view of evidence that the onset of anxiety symptoms typically precedes that of hazardous alcohol use [15], the co-occurrence of anxiety and alcohol use is often explained by the self-medication model [17,18], whereby alcohol is consumed in an attempt to reduce or cope with anxiety symptoms. Over time, this can lead to progressively greater alcohol intake, related psychosocial problems, functional impairment, and heightened stress and anxiety [19,20]. Among young people, overly positive expectancies about the tension-reducing and social lubricant effects of alcohol are common, and anxious young people may be particularly susceptible to the use of alcohol to cope with symptoms of anxiety and nervousness as they navigate new social and occupational challenges [21,22]. Indeed, coping-motivated drinking has been linked to the development of alcohol-related problems [23-25], and anxiety disorders are associated with an earlier first use of alcohol [26] and increased risk of progression from first alcohol use to regular use and from regular use to alcohol use disorder [27]. If left untreated, anxiety and hazardous alcohol use tend to fuel each other in an exacerbating feed-forward cycle that interferes with recovery from either condition [28-31].

### Integrating Anxiety and Alcohol Use Interventions Improves Outcomes

People with co-occurring anxiety and hazardous alcohol use tend to respond poorly to standard, single-disorder interventions [32-34]. Increasingly, the co-occurrence of anxiety symptoms and hazardous alcohol use is understood as a clinically important relationship involving mutually reinforcing connections that are likely to require integrated interventions [16,35]. Evidence from adult samples [30,36,37] suggests integrating treatment for co-occurring anxiety and alcohol use disorders is a promising approach that is intuitively appealing to patients. Our research group has developed an integrated cognitive behavioral therapy (CBT) for co-occurring social anxiety and alcohol use disorders [28], which has demonstrated greater improvements in anxiety symptoms, depression, and overall functioning compared with standard treatment approaches [37]. Furthermore, our work with young adolescents (aged 13-14 years) has demonstrated that early intervention implemented in secondary schools to improve coping with high-risk personality traits, including anxiety sensitivity, effectively reduces alcohol misuse and alcohol-related harms over a 3-year period [38]. Most recently, a randomized controlled trial (RCT) of a youth-focused, internet-delivered, early intervention targeting co-occurring depression and harmful alcohol use has demonstrated clinically significant short-term symptom improvements compared with an attention control among young adults [39]. These findings demonstrate the clear clinical benefits of integrated approaches and demonstrate that age-appropriate early intervention has the potential to prevent or halt the escalation of anxiety and drinking into disorder-level problems.

### The Inroads Program: Providing Accessible, Age-Appropriate, Early Intervention for Young Adults

The increased availability and opportunities for drinking during early adulthood, combined with the peak in onset of anxiety disorders, suggests this is a critical developmental window for interrupting the trajectory into chronic anxiety and alcohol use disorders. Despite the associative links between anxiety and hazardous alcohol use, there are no existing youth-focused interventions that target anxiety symptoms, hazardous alcohol use, and the interconnections between them. The internet presents a promising delivery method for this age group as it reduces barriers to seeking treatment such as fear of judgment or stigma, cost, and difficulties accessing treatment [14]. Reviews suggest young people prefer internet-delivered over face-to-face treatments [40], and limitations relating to engagement and retention can be ameliorated through the provision of therapist support via phone, chat, or email [41]. To meet this need for an age-appropriate, internet-delivered, integrated intervention targeting anxiety and hazardous alcohol use, the *Inroads* program was developed. The program was designed in consultation with the target age group to ensure the content and features (ie, case vignettes, skill-based videos, language, illustrations, and layout) were deemed relevant, acceptable, and appealing and would maximize engagement of anxious young people at risk of hazardous alcohol use. This brief, 5-session intervention combines therapist phone and email support with internet-delivered CBT content via contemporary Web design, youth-focused illustrations, videos, and vignettes. The active intervention components draw from our integrated CBT program for adults with social anxiety and alcohol use disorder [28] and our Web-based youth program for alcohol use and depression treatment [39]. The *Inroads* program and development process are described in full elsewhere [42].

### Aims

This study will evaluate the efficacy of the *Inroads* program—a brief, Web-based CBT intervention that combines 5 online skills-based modules with minimal psychologist support for young adults (aged 17-24 years) who experience both anxiety symptoms and hazardous or harmful alcohol use. The study will be an RCT comparing the *Inroads* program with a control condition who will receive assessment plus alcohol guidelines and information. The study will be the first to evaluate the benefits of an early intervention for young adults targeting hazardous alcohol use, anxiety symptoms, and the interconnections between these problems.

It is hypothesized that compared with the control condition, the *Inroads* intervention will achieve greater reductions in (1) alcohol use, including drinks per drinking day and frequency of binge drinking; (2) alcohol-related harms; and (3) anxiety symptoms at 8 weeks post baseline. To assess the sustainability of these effects, outcomes for both groups will be assessed again at 6 months post baseline. Furthermore, it is hypothesized that participants allocated to receive the *Inroads* program will report decreased coping-motivated drinking and positive alcohol expectancies and greater improvements from baseline to post intervention on secondary outcomes of stress, social anxiety and depression symptoms, overall functioning, and quality of life.

## Methods

### Study Setting and Design

**Figure 1:**
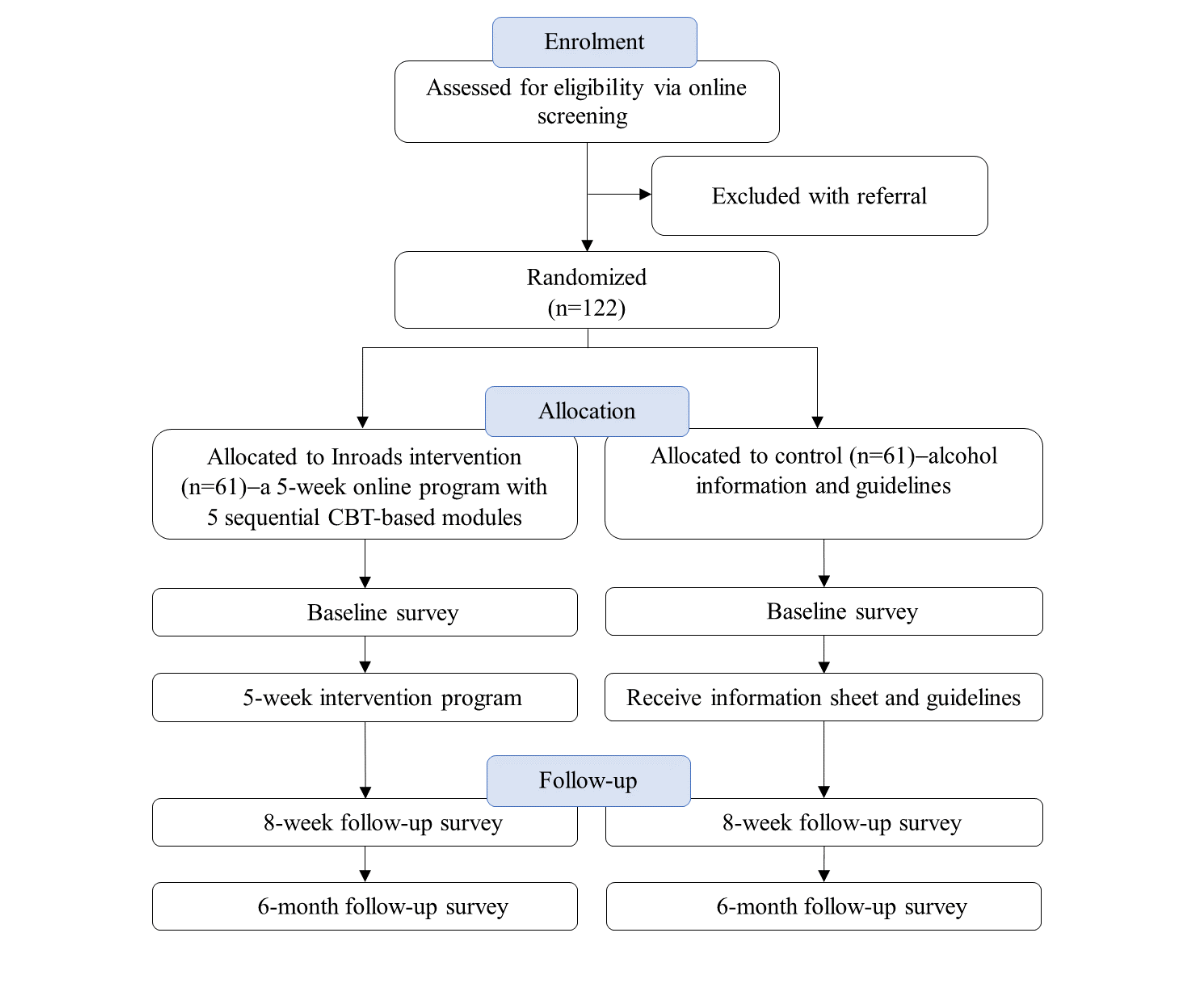
Inroads study design. CBT: cognitive behavioral therapy.

The study will be conducted nationally across Australia and involves a parallel RCT in which eligible participants will be individually randomized into either (1) the intervention condition (*Inroads* program) or (2) the control condition (assessment plus alcohol guidelines and information). The primary end point will be the posttreatment assessment, conducted at 8 weeks following baseline, with a secondary end point at 6 months after baseline. Figure 1 depicts the study design.

### Ethics Approval and Registration

The study has been prospectively registered with the Australian New Zealand Clinical Trials Registry (ACTRN12617001609347) and received ethical approval from the University of New South Wales Human Research Ethics Committee (HC17185). Informed consent will be obtained electronically from all participants and confidentially assured via rigorous data encryption.

### Participants

A total of 122 participants (male and female) aged between 17 and 24 years who report anxiety symptoms and hazardous or harmful alcohol consumption will be recruited into the study in 2018. This age range captures the transition from late adolescence at 17 years into the unique developmental stage of young adulthood [43].

#### Recruitment

Participants will be recruited through a variety of advertising methods including media coverage (TV and radio), social media posts, paid online advertising via social media and search engines (eg, Facebook, Twitter, Instagram, YouTube, and Google search), distribution of posters and flyers at universities, technical and further education institutes and youth-focused institutions, and referral from youth mental health services. Potential participants will be referred to the study website [44], which provides a description of the study. To be assessed for the study, interested participants will click a link to provide informed consent before proceeding to the online eligibility assessment.

### Eligibility Criteria

Individuals who consent to participate will be directed to complete a 15-min online eligibility assessment. Inclusion and exclusion criteria are shown in Textboxes 1 and 2, respectively. Eligible participants will be invited to complete an online baseline assessment before proceeding with the trial. Participants who do not meet eligibility criteria will be provided with a list of referral options.

**Inclusion criteria.**

- Aged between 17 and 24 years.
- Living in Australia.
- Currently reporting hazardous or harmful levels of alcohol use, as indicated by an Alcohol Use Disorders Identification Test score ≥8 [45].
- Experiencing at least mild symptoms of an anxiety disorder, as indicated by a score ≥5 on the Generalized Anxiety Disorder-7 (GAD-7) Questionnaire [46] or a score ≥6 on the Mini-Social Phobia Inventory (Mini-SPIN) [47]. Although the Mini-SPIN focusses on anxiety symptoms consistent with social anxiety disorder, the GAD-7 has been found to index symptoms across multiple disorders and is sensitive to GAD, panic, and social anxiety disorder, facilitating screening of young people presenting with a range of anxiety presentations [48].

**Exclusion criteria.**

- Inability or unwillingness to provide contact information.
- Insufficient English literacy.
- Inability to access the internet to participate in the program.
- Daily use of cannabis or benzodiazepines or weekly use of psychostimulants (assessed by the National Institute on Drug Abuse quick screen questions [49]).
- Primary current concern as identified by the participant is related to trauma symptoms or substance use other than alcohol.
- Significant risk of complicated alcohol withdrawal (indicated by past experience of severe alcohol withdrawal symptoms such as seizures, hallucinations, or high fever).
- Active suicidal ideation (indicated by a single item assessing experience of suicidal thoughts and intent in the past 2 weeks).
- Active symptoms of psychosis (score ≥3 on the Psychosis Screening Questionnaire [50]) or currently accessing ongoing psychological treatment for mental health or drug or alcohol problems.

### Allocation

To avoid bias, participants will be individually randomized to either the intervention or control condition via the trial website using a computer-generated randomization sequence, which is concealed from the investigators. Automatic randomization within the Web-based program removes the potential for researcher involvement. Randomization will occur directly after completion of the online baseline assessment. Following randomization, the study psychologists (LS, EK, and AB) and project coordinator (KP) will be informed of group allocation to deliver the intervention (phone or chat sessions and motivational emails) to participants allocated to the intervention condition, whereas the research assistant (BL) who is responsible for reminding participants to complete follow-up assessments will remain blind to allocation status.

### Assessments

All assessments are well-validated instruments commonly used in mental health and alcohol research and will be administered online via the trial website hosted by a secure, dedicated server in Australia. Participants will complete an online eligibility screening and baseline assessment before random allocation. Automatic email prompts to complete follow-up assessments will be sent to participants at 8 weeks (primary end point) and 6 months (secondary end point) after baseline. The following evidence-based strategies will minimize data attrition [51,52]: (1) monetary incentives (Aus $30 gift voucher) for each follow-up assessment completed, (2) collection of multiple sources of contact information at baseline (eg, email, mobile number, and postal address), (3) user-friendly electronic survey design that can be completed via multiple devices (eg, via mobile phone), (4) personalized reminder messages (short message service [SMS] text message and email) to complete survey, and (5) follow-up letter with photo of the research team and telephone call from the research assistant (blind to allocation status) to those participants who do not respond. Self-report assessment, use of standardized reminder templates, and blinding of the research assistants administering follow-up calls removes the potential for researcher influence over study results. The timing of assessments is detailed in the *Inroads* study schedule in Table 1.

### Measures

Primary and secondary outcomes of the study were assessed at baseline, 8-weeks post baseline, and 6-months post baseline using validated psychometric instruments.

#### Primary Outcomes

Due to the focus of the study on co-occurring anxiety and hazardous alcohol use, primary outcomes encompass measurement across both anxiety symptoms and alcohol-related problems. Primary outcomes for alcohol are consumption per drinking day, calculated from the total number of Australian standard drinks (10 g of alcohol) consumed in the past month and assessed by a computerized version of the Timeline Follow-Back (TLFB; [53,54]) procedure and alcohol-related harm as assessed by the Brief-Young Adult Alcohol Consequences Questionnaire (B-YAACQ; [55]). The TLFB is a widely used procedure that has been validated across a number of countries and among a variety of subpopulations, including young adults, and the self-report computerized version has demonstrated good reliability with test-retest correlations exceeding 0.85 (see review by Deady [56]). The B-YAACQ assesses 24 age-appropriate consequences of alcohol consumption in the past 30 days using a dichotomous (0 “no” or 1 “yes”) response format. Scores range from 0 to 24, with higher scores indicating more problems and negative consequences from drinking. Primary outcomes for anxiety will be assessed by the Generalized Anxiety Disorder-7 (GAD-7) [48], which is sensitive to symptoms across anxiety disorders and has been previously used to assess outcomes in trials of internet-delivered treatment for mixed anxiety samples [57]. The GAD-7 contains 7 items, ranging from 0 “not at all” to 3 “nearly every day.” Total scores range from 0 to 21, with scores of 5, 10, and 15 representing cut points for mild, moderate, and severe anxiety, respectively.

**Table 1:** *Inroads* study timeline.

Assessments		Study period				
		Enrollment	Pre-allocation	Allocation	Post-allocation	
			t_1_ (baseline)		t_2_ (8 weeks)	t_3_ (6 months)
**Enrollment**						
	Eligibility screen	✓^a^	–^b^	–	–	–
	Informed consent	✓	–	–	–	–
	Allocation	–	–	✓	–	–
**Intervention**						
	*Inroads* ^c^	–	–	✓	✓	–
	Control	–	–	✓	✓	–
**Assessments**						
	Alcohol Use Disorder Identification Test	✓	–	–	✓	✓
	Generalized Anxiety Disorder-7	✓	–	–	✓	✓
	Mini-Social Phobia Inventory	✓	–	–	–	–
	National Institute on Drug Abuse quick screen	✓	–	–	–	–
	Psychosis Screening Questionnaire	✓	–	–	–	–
	Alcohol Outcome Expectancies tension-reduction scale	–	✓	–	✓	✓
	Brief Young Adult Alcohol Consequences Questionnaire	–	✓	–	✓	✓
	Depression and Anxiety Stress Scale	–	✓	–	✓	✓
	Drinking Motives Questionnaire-Revised	–	✓	–	✓	✓
	Emotion Regulation Questionnaire	–	✓	–	✓	✓
	Life Events Checklist for Diagnostic and Statistical Manual of Mental Disorders, Fifth Edition (DSM-5)	–	✓	–	✓	✓
	Post-Traumatic Stress Disorder symptom checklist for DSM-5	–	✓	–	✓	✓
	Self-Compassion Scale-Short Form	–	✓	–	✓	✓
	Sheehan Disability Scale	–	✓	–	✓	✓
	Social Phobia Scale and Social Interaction Anxiety Scale-Short forms	–	✓	–	✓	✓
	Substance Use Risk Profile Scale	–	✓	–	✓	✓
	Treatment Acceptability Questionnaire	–	–	–	✓	–
	Timeline Follow-Back	–	✓	–	✓	✓
	Working Alliance Inventory-Short Form	–	–	–	✓	–

^a^Indicates that enrollment, intervention delivery and/or assessments occurred at these time points.

^b^Indicates that enrollment, intervention delivery and assessments were not relevant at these time points.

^c^*Inroads* intervention includes 5 modules which are delivered over 5 weeks, with flexibility in module completion provided up until t_2_ (8-week post- baseline assessment).

#### Secondary Outcomes

Module completion, time spent, rate of completion, and number and duration of therapist contacts via email, chat, and phone will be recorded to measure treatment retention and dose. Frequency of binge drinking (past month consumption of ≥5 standard drinks on 1 occasion) will be calculated from alcohol consumption data collected using the TLFB procedure [53,54]. Symptoms specific to social anxiety will be assessed using the 12-item Social Phobia Scale and Social Interaction Anxiety Scale-short forms [58]. Items are scored on a 5-point Likert-type scale from 0 “Not at all characteristic or true of me” to 4 “Extremely characteristic or true of me.” Total scores range from 0 to 48, with higher scores indicating higher levels of social anxiety. Symptoms of anxious arousal and depression will be assessed using the 21-item anxiety and depression subscales of the Depression and Anxiety Stress Scale [59]. Items are rated on a 4-point scale (0-3), summed and doubled for each subscale. Subscale scores range from 0 to 42, with higher scores indicating greater severity of emotional symptoms. Overall, functional impairment and quality of life will be assessed by the Sheehan Disability Scale [60] *.* Participants rate the extent to which their work, social life/leisure activities, and home life/family responsibilities are impaired by their symptoms on a 10-point visual analog scale. Higher numbers indicate greater impairment. The 3 items may be summed into a single dimension of global functioning impairment that ranges from 0 “unimpaired” to 30 “highly impaired.” The 28-item Drinking Motives Questionnaire-Revised [61] will be used to assess alcohol use motives across 5 subscales: social, coping-anxiety, coping-depression, enhancement, or conformity. Each item is rated on a 5-point Likert scale ranging from 1 “almost never/never” to 5 “almost always/always.” Subscales are scored as the average across the items within a scale, which allows a direct comparison across subscales. Positive alcohol expectancies will be assessed by the 17-item alcohol tension-reduction expectancies scale [62]. Each item is scored from 0 “not at all important” to 3” very important,” with total scores ranging from 0 to 51. Higher scores indicate greater expectancies regarding the tension-reducing effects of alcohol.

Additional measures will be included to explore secondary research questions, namely (1) Emotion Regulation Questionnaire [63] to assess emotional regulation difficulties; (2) Self-Compassion Scale-Short Form to assess self-compassion [64]; (3) Life Events Checklist for Diagnostic and Statistical Manual of Mental Disorders, Fifth Edition (DSM-5) [65], adapted to assess lifetime exposure to potentially traumatic events; (4) Post-Traumatic Stress Disorder (PTSD) symptom checklist [66] to assess severity of past-month PTSD symptoms according to DSM-5; (5) Working Alliance Inventory-Short Form-revised [67] to assess therapeutic alliance; (6) Treatment Acceptability Questionnaire [68]; and (7) Substance Use Risk Profile Scale [69] to assess personality risk factors. Demographic information including age, sex, education, employment, country of birth, sexuality, geographical location, and treatment use (ie, medication and treatment from health professionals such as psychologists, psychiatrists, counselors, and general practitioners) will also be collected.

#### Inroads Anxiety and Alcohol Use Intervention

The *Inroads* program is a therapist-supported, internet-delivered CBT program aimed at reducing symptoms of anxiety, hazardous alcohol consumption, and alcohol-related harms. It involves 5 sequential modules over a 5-week period that focus on enhancing motivation to change and developing CBT strategies to manage anxiety and hazardous alcohol use. Each module should take approximately 30 min to 45 min to complete, and to allow time for skill practice and consolidation, new modules will become available at a rate of 1 module each week, irrespective of whether the previous module has been completed. Automated email and SMS text message reminders to complete program modules will be provided weekly. Goal setting, planning, and review are completed each module, with new skills being introduced in each module (Table 2).

The content for each module is delivered via written text, images, infographics, and interactive forms, whereby participants are guided to identify their goals recognize their cognitive and/or behavioral responses, and practice CBT skills by working through personal examples. Additional online forms are provided for homework practice. In modules 2, 3, and 4, a brief 3-min animated video illustrates the key skills introduced in the module (module 2: realistic thinking, module 3: strategies to reduce or avoid drinking, module 4: facing fears to overcome anxiety). In addition, as participants work through the program, they follow the stories of 2 characters. This narrative is presented via audio segments (with accompanying text) to illustrate case examples aligned with the key concepts or skills in each module. A 5-item quiz at the end of each module provides the opportunity for participants to test and reinforce their knowledge of the key points.

Therapist support will be provided by a team of trained psychologists, ranging from recent graduates to experienced clinicians, who will receive supervision from experienced clinicians. At the completion of each module, participants will receive an email providing feedback, troubleshooting, and personalized suggestions aligned to module content. In addition, telephone and/or chat sessions following modules 1 (30 min) and 4 (30 min) will focus on motivational enhancement, developing a shared anxiety-drinking problem formulation, troubleshooting, and tailoring behavioral experiments and cognitive therapy exercises. The 5 modules are intended to be completed weekly; however, to allow some flexibility in the rate of completion, postintervention surveys will be administered to all participants 8 weeks after baseline. Participants in the intervention will also be asked to complete another follow-up survey 6 months post baseline.

**Table 2:** New skills introduced in each module of the *Inroads* program.

Modules	Skills learned
Module 1	Normative feedback about alcohol use; understanding motives for change and the interrelationship between anxiety and alcohol use; psychoeducation regarding the cognitive, physiological, and behavioral aspects of anxiety and alcohol use; goal setting and drinking limits; and emotion surfing to ride out cravings and uncomfortable feelings
Module 2	Understanding the ABC^a^ model and cognitive therapy targeting anxious thoughts
Module 3	Cognitive behavioral therapy strategies for sticking to drinking limits, cognitive therapy targeting positive alcohol expectancies (ie. ‘drinking thinking’), assertiveness, and handling group dynamics
Module 4	Understanding avoidance and anxiety and graded behavioral experiments
Module 5	Enhancing social support, longer-term goal setting, and relapse prevention

^a^ABC: Refers to activating event or objective situation, belief, and consequences.

#### Assessment and Alcohol Information Control

Participants in control condition will receive assessment followed by an online information pamphlet outlining the effects of alcohol and risks of overuse, the Australian National Health and Medical Research council’s recommended guidelines for alcohol consumption, and a list of links to national telephone helplines and alcohol information websites. The information pamphlet will be available for immediate download and will also be emailed to them. Past research demonstrates that answering detailed questions about drinking alters subsequent self-reported behavior, particularly among young adult samples [70-72], and thus, it is expected that the assessment procedures in combination with information provision will lead to a modest decrease in alcohol consumption in the control group. By using an alcohol-only control condition, the *Inroads* integrated intervention can be compared with current recommendations for comorbidity management, which state that alcohol use problems should be addressed before co-occurring conditions such as anxiety [73]. Participants in the control group will be informed that they will be recontacted in 8 weeks and 6 months for follow-up surveys, and after completion of the final survey, they will be offered the *Inroads* program.

### Safety Protocol

Before consenting to the study, the information statement will provide all participants with a list of crisis and support services. At entry to the study, participants will be screened for risk of complicated alcohol withdrawal and active suicidal ideation in the past 2 weeks, and those who screen positive will be referred to appropriate support services. For participants allocated to the *Inroads* program, the project psychologists will monitor symptoms during phone, email, and/or chat contact and via review of content submitted online. The Web-based program will provide links for participants to request additional support from the project psychologists via phone or chat room. Psychologists will provide up to a maximum of 2 hours of additional phone and/or chat support where required, and in cases where a severe deterioration or safety risk is indicated, participants will be referred to appropriate services for more intensive support. Participants in both conditions will complete assessments 8 weeks and 6 months after baseline, which provides an opportunity for symptom monitoring, and all participants will be provided with a list of support services that can be accessed should they require additional help. In addition, the trial website will automatically detect any English words or phrases entered in the program modules or assessment surveys that are consistent with the vernacular of suicidal ideation [74]. If any words indicative of suicidality are detected, a notification will be sent to the project psychologists, who will manually examine the relevant content. When manual review suggests suicidal ideation, the psychologist will contact the participant to conduct a risk assessment and provide referral to appropriate support services.

### Sample Size

Previous evaluations of brief, multisession, internet-delivered interventions for young adults have indicated an effect size at post intervention of between 0.68 and 0.99 for reduction in the number of drinks consumed [39,75], 0.56 for reduction in alcohol-related consequences [75], and 0.59 for reduction in anxiety symptoms [76]. Optimal Design software [77] was used to calculate the required sample size, taking into account the multilevel analysis with a nested repeated measures design. Using a conservative approach with intraclass correlation coefficient of .55 estimated based on our adult comorbidity trial [37], power calculations indicated a sample size of 90 (45 each group) would be required to detect a moderate effect size of 0.50 between the intervention and control group with power=0.8 and alpha=.05. To allow for data attrition at post assessment, estimated at 35% (n=32/90) [78], a total sample of 122 individuals will be recruited to the study.

### Statistical Analysis

Primary analyses will use multilevel mixed effects analysis for repeated measures [79,80], which is a flexible analytic approach for modeling change over time that has emerged as a flexible and rigorous method for analyzing RCT results. The approach has a number of advantages over traditional approaches, including better treatment of missing data and flexible modeling of variance at the individual level, time effects, and the within-subject covariance structure [79,80]. Models will include a random intercept, and preliminary models will be estimated and model fit statistics examined to determine the most appropriate model and covariance structure. All models will use baseline measurements as the reference point to estimate participant-specific starting points and change over time. Intervention condition will be represented by a dummy-coded variable, and the condition by time interaction will be examined to assess between-group differences in treatment response over time. Treatment dose (hours of therapist contact and module completion) will be entered as an independent variable in secondary analyses to examine moderation of treatment effects. For outcomes with evidence of significant intervention effects, Cohen *d* will be calculated from model estimated marginal means and standard errors to determine the size of effect between conditions at the relevant end point. All analyses will be carried out on an intention-to-treat basis, retaining and analyzing all participants in the intervention groups they were originally allocated to. Missing data will be accounted for in all analyses using maximum likelihood estimation, and the impact of missing data on study conclusions will be examined using recommended methods [81]. Data analyses will be conducted using Stata, version 15 [82], with significance levels set at *P*<.05 (95% CI).

## Results

The study is funded from 2017 to 2020 by Australian Rotary Health. Recruitment is expected to be complete by late 2018, with the 6-month follow-ups to be completed by mid-2019. Results are expected to be submitted for publication in 2020.

## Discussion

### Potential Findings

There is a need for evidence to guide policy and practice to address hazardous alcohol use occurring in the context of anxiety symptoms. We describe the protocol for the first trial of a youth-focused, Web-based, early intervention that targets anxiety, hazardous alcohol use, and the mutually reinforcing connections between these problems. Given the transition into early adulthood is a key risk period for the onset of anxiety and alcohol use disorders [2-5], evidence of improved outcomes would provide a scalable intervention strategy to reduce the substantial burden, social costs, and disability associated with these disorders.

### Strengths and Limitations

The study will address an important knowledge gap by examining for the first time the benefits of an integrated, Web-based, early intervention approach that targets the interrelationship between anxiety and alcohol use. An additional strength of this study is the intervention delivery in a brief format that has been adapted and designed specifically for young adults, featuring engaging and interactive content delivery, visually appealing illustrations and videos, and age-appropriate case vignettes. Delivery via the internet is a further strength, given that young people report a preference for internet-delivered over face-to-face treatments [40], and this format circumvents some of the barriers to youth seeking treatment, including fear of judgment or stigma, and difficulties accessing treatment at a convenient time or location [14].

A challenge for this study is treatment retention and data attrition. Motivation to change alcohol consumption is known to fluctuate over time [83], and treatment dropout is relatively high, with a recent meta-analysis estimating dropout from substance use treatment at 36.4% [78]. Lower attrition is typically observed for anxiety disorders (19.6%; [78]); however, higher treatment dropout is typical in samples with comorbid problems [84-86]. Although internet-delivered mental health interventions offer a number of advantages, particularly for young people, they are also associated with higher dropout rates (34.2%) compared with face-to-face treatment delivery (24.6% and 25.1% for individual and group treatment dropout rates, respectively; [78]). To address this potential limitation, the *Inroads* program was developed to combine internet-delivered content with psychologist support via email and phone. In a systematic review, supplementing internet-delivered interventions with support was associated with improved outcomes and greater treatment retention [41]. Furthermore, therapeutic components of the *Inroads* program are consolidated within a brief, 5-session intervention, in view of evidence that time pressure is a common barrier to treatment [87] and young people are less likely to commit to longer treatment duration [88]. To minimize data attrition, we will implement a comprehensive follow-up protocol incorporating multiple evidence-based strategies [51,52], including monetary incentives and multiple reminder messages, that we have used to good effect in previous trials involving young people [38,39]. Moreover, missing data will be accounted for in all analyses using maximum likelihood estimation, and the impact of missing data on study conclusions will be examined using recommended methods [81]. A second limitation of the study is that the control condition is not matched to the *Inroads* program in terms of length of intervention content or access to therapist support. Nonetheless, the comparison condition involves comprehensive alcohol assessment and information provision, which has been found to alter subsequent self-reported drinking, particularly among young adults [70-72], and thus provides a suitable benchmark to assess the additive benefits of the *Inroads* program. We consider the comparison between this integrated approach and single-disorder interventions of equivalent length an important next step should the program prove efficacious in this trial.

### Conclusions and Implications

The link between anxiety and hazardous alcohol use is well established, and when these conditions do co-occur, the presentation tends to be more severe and difficult to treat; indeed, psychiatric comorbidity represents one of the most significant challenges to effective treatment provision and an urgent global priority for health research. The increased availability and opportunities for drinking during early adulthood, combined with the peak in onset of anxiety disorders, suggests this developmental window represents a promising opportunity for early intervention to interrupt the trajectory toward co-occurring anxiety and alcohol use disorders. By explicitly addressing the expectancies and behavioral links between anxiety and alcohol use as well as enhancing CBT coping skills specifically tailored to the unique stressors and drinking contexts of young adulthood, the *Inroads* program has the potential to improve anxiety symptoms and reduce alcohol consumption before these problems become entrenched. The Web-based format of the program combined with minimal therapist support via phone or email means that if effective, the program could be widely disseminated, with potential to maximize impact by reaching young people who are not currently able or willing to access face-to-face treatment.

## Abbreviations

B-YAACQ: Brief-Young Adult Alcohol Consequences Questionnaire
CBT: cognitive behavioral therapy
DSM-5: Diagnostic and Statistical Manual of Mental Disorders, Fifth Edition
GAD-7: Generalized Anxiety Disorder-7
PTSD: Post-Traumatic Stress Disorder
RCT: randomized controlled trial
SMS: short message service
TLFB: Timeline Follow-Back
